# Does Sleep Moderate the Effects of Exercise Training or Complex Mental and Social Activities on Cognitive Function in Adults With Chronic Stroke? Secondary Analysis of a Randomized Trial

**DOI:** 10.1093/gerona/glae264

**Published:** 2024-11-08

**Authors:** Ryan S Falck, Ryan G Stein, Jennifer C Davis, Janice J Eng, Laura E Middleton, Peter A Hall, Teresa Liu-Ambrose

**Affiliations:** Aging, Mobility and Cognitive Health Laboratory, Faculty of Medicine, Department of Physical Therapy, University of British Columbia, Vancouver, British Columbia, Canada; Aging, Mobility and Cognitive Health Laboratory, Faculty of Medicine, Department of Physical Therapy, University of British Columbia, Vancouver, British Columbia, Canada; Applied Health Economics Laboratory, Faculty of Management, University of British Columbia – Okanagan Campus, Kelowna, British Columbia, Canada; Neurorehabilitation Research Program, GFS Rehabilitation Centre, Faculty of Medicine, Department of Physical Therapy, University of British Columbia, Vancouver, British Columbia, Canada; Department of Kinesiology and Health Sciences, University of Waterloo, Waterloo, Ontario, Canada; School of Public Health Sciences, University of Waterloo, Waterloo, Ontario, Canada; Aging, Mobility and Cognitive Health Laboratory, Faculty of Medicine, Department of Physical Therapy, University of British Columbia, Vancouver, British Columbia, Canada

**Keywords:** Cognitive function, Environmental enrichment, Exercise training, Sleep, Stroke

## Abstract

**Background:**

Exercise (EX) or cognitive and social enrichment (ENRICH) are 2 strategies for promoting cognition poststroke. Whether sleep moderates the effects of EX or ENRICH on cognition in adults with chronic stroke is unknown.

**Methods:**

A 3-arm parallel randomized clinical trial among community-dwelling adults aged 55+ years with chronic stroke (ie, ≥12 months since stroke). Participants were randomized to 2× per week EX, ENRICH, or balance and tone control (BAT). At baseline, device-measured sleep duration and efficiency were measured using wrist-worn actigraphy; self-reported quality was measured by Pittsburgh Sleep Quality Index (PSQI). Participants were categorized at baseline as having good or poor device-measured duration, device-measured efficiency, or self-reported quality based on PSQI. The primary cognitive outcome was Alzheimer’s Disease Assessment Scale Plus (ADAS-Cog-Plus) measured at baseline, 6 months (end of intervention), and 12 months (6-month follow-up). We examined if baseline sleep categorizations (ie, good vs poor) moderated the effects of EX or ENRICH on ADAS-Cog-Plus.

**Results:**

We enrolled 120 participants in the trial (EX = 34; ENRICH = 34; BAT = 52). Sleep quality (ie, device-measured sleep efficiency or self-reported sleep quality) categorization moderated effects of EX (but not ENRICH) on ADAS-Cog-Plus. Compared with BAT participants with poor sleep quality, EX participants with poor sleep quality had better ADAS-Cog-Plus performance at 6 months (estimated mean difference for those with poor device-measured sleep efficiency: −0.48; 95% CI [−0.85, −0.10]; *p* = .010); estimated mean difference for those with poor self-reported sleep quality: −0.38; 95% CI [−0.70, −0.07]; *p = *.014). There was no effect of EX on ADAS-Cog-Plus for participants with good sleep quality. Device-measured sleep duration did not moderate intervention effects.

**Conclusions:**

Exercise is particularly beneficial in improving cognitive function in adults with chronic stroke and poor sleep quality.

Worldwide, someone suffers a stroke every 40 seconds (1). A stroke doubles one’s risk for dementia (2), and stroke-related cognitive deficits are associated with reduced functional independence, institutionalization, reduced quality of life, and early death (3). Stroke survivors thus need targeted interventions to promote cognitive function and reduce the risk of dementia. Two promising strategies for promoting cognitive health in stroke survivors are (1) exercise training (EX) and (2) cognitive and social enrichment (ENRICH) (4).

Exercise training is defined as planned or structured physical activity with the intent of increasing or maintaining physical fitness (5). Although the precise prescription of EX (ie, type, intensity, or volume) for enhancing cognitive function is still uncertain, evidence from randomized clinical trials (RCTs) suggests that moderate-or-higher intensity EX improves cognitive function and promotes better brain health among older adults (6).

Cognitive and social enrichment is broadly an intervention designed to increase cognitive and social activity by provision of a stimulating environment (7). The premise of this strategy is that engaging in both cognitive and social activities can stimulate higher-order cognitive processes (eg, memory and executive function) and thus promote better cognitive function. A systematic review by Kelly and colleagues (8) highlighted the importance of social relationships for maintaining cognitive function as adults age. A meta-analysis by Hill and others (9) determined that cognitive training had a moderate-sized effect on cognitive function in adults at risk for cognitive decline.

Despite the high prevalence of cognitive deficits and increased risk for dementia among adults with stroke, only a few studies have examined the effects of EX on cognitive function in people with chronic stroke (10–12). A RCT of 38 adults with chronic stroke (ie, ≥6 months postischemic stroke) found that 8 weeks of aerobic EX improved processing speed compared with 8 weeks of stretching (10). Moore and colleagues (11) conducted a RCT of 40 adults with chronic stroke, which determined that 19 weeks of thrice-weekly multimodal EX improved general cognition and increased cerebral blood flow compared with resistance EX. Conversely, a secondary analysis of a RCT among 50 adults with chronic stroke found that 6 months of high-intensity aerobic EX did not significantly improve cognitive function relative to a low-intensity balance and tone control (BAT) group (12).

Much less is known about the effects of ENRICH on cognitive function in people with chronic stroke. Briefly, a 6-month pre-post study determined that twice-weekly multimodal EX combined with once-weekly ENRICH improved cognitive function in people with chronic stroke (13). A pilot RCT compared this same multicomponent intervention to a wait-list control and found benefits to aspects of cognitive and physical function (14).

Previously, we investigated the effects of twice-weekly moderate-intensity EX or ENRICH compared with a BAT group on cognitive function in older adults with chronic stroke (4). We showed that EX significantly improved cognitive function in adults with chronic stroke; we did not find a significant effect of ENRICH. However, a large degree of variation exists in the efficacy of nonpharmacological interventions to promote cognitive function such as EX or ENRICH (15). For example, a meta-analysis determined that effect size estimates for the impact of EX on cognitive function varied between a Hedge’s *g* of −1.89 and 3.41 (15). Thus, we need to identify key moderators of interventions to promote cognitive health in order to determine for whom these types of interventions are most beneficial (16,17).

Sleep may be one key moderator of the effects of EX or ENRICH interventions on cognitive function. Poor sleep is common following stroke (18). Approximately 40% of stroke survivors have diagnosed sleep disorders such as excessive daytime sleepiness, obstructive sleep apnea (OSA), and insomnia (19,20). Poor sleep quality among stroke survivors increases the risk of recurrent stroke by threefold and the risk of early death by 76% (21). Of relevance, adults with chronic stroke (ie, ≥12 months since stroke sequelae) and poor sleep also experience larger deficits in cognitive performance compared with their counterparts without sleep problems (22). It is thus plausible that older adults with chronic stroke and poor sleep may be more likely to have improved cognitive performance from nonpharmacological therapies to promote cognition.

Whether sleep moderates the effects of EX or ENRICH on cognitive function in adults with stroke has not been examined. However, there is some evidence to suggest that sleep moderates the effects of EX on cognitive function (23,24). A narrative review concluded that the effects of a single-bout of EX on cognitive function were more robust in older adults with better sleep (23). In contrast, a secondary analysis of a 6-month RCT determined that the effect of EX on cognitive function was greater in cognitively healthy older adults with poor self-reported sleep (24). Importantly, the moderating effect of sleep was evident even though there was no effect of the EX intervention on sleep. These results suggest EX-induced changes in sleep are not necessary for EX-induced changes in cognitive function. To our knowledge, no study has examined whether sleep moderates the effects of ENRICH on cognitive function.

Thus, in this secondary analysis, we examined whether baseline sleep quantity or quality moderates the effects of EX or ENRICH on cognitive function in adults with chronic stroke. We hypothesized that baseline sleep quantity and quality would moderate the effects of EX and ENRICH on cognitive function. Specifically, EX and ENRICH would each improve cognitive function among those with poor sleep but not among those with good sleep.

## Method

### Study Design

**Figure 1.**
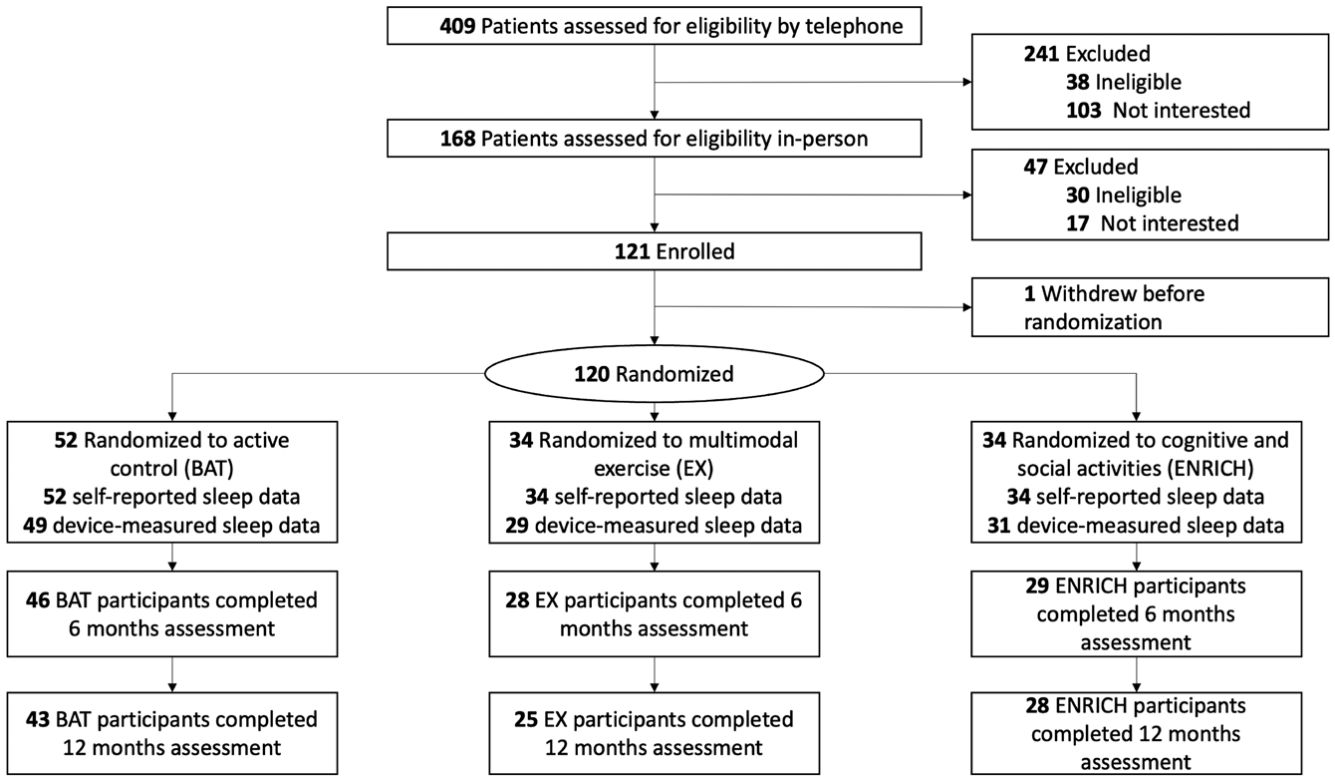
CONSORT diagram.

This was a secondary analysis of a 3-arm parallel, single-blinded, 6-month RCT with a 6-month follow-up in a research center (Vancouver, British Columbia, Canada) to examine the effects of EX or ENRICH activities on cognitive function in community-dwelling adults with chronic stroke (4). Participants were measured at baseline, at the end of the 6-month intervention, and at 6-month follow-up (Figure 1). Ethical approval was provided by University of British Columbia’s Clinical Research Ethics Board (H13-00715) and Vancouver Coastal Health Research Institute (V13-00715). The trial protocol and the primary study results are published (ClinicalTrials.gov identifier: NCT01916486) (4,25).

### Recruitment

Participants were recruited from the community as well as from stroke clinics. Enrollment and randomization occurred from June 6, 2014 to February 26, 2019 (4).

### Inclusion and Exclusion Criteria

We included community-dwelling adults who had an ischemic or hemorrhagic stroke. Additional inclusion criteria were: (1) aged ≥55 years; (2) a history of a stroke ≥12 months prior to study enrollment; (3) a Mini-Mental State Examination (MMSE) (26) score of ≥20/30 at screening, including a perfect score on the 3-step command to ensure intact comprehension and ability to follow instructions; (4) English speaking; (5) not expected to start or were on a stable fixed dose of cognitive medications during the study period; (6) able to walk 6 m with rest intervals with or without assistive devices; and (7) not currently participating in any regular therapy or progressive EX. Exclusion criteria were neurodegenerative disease, dementia, at high risk for cardiac complications during EX, taking medications that may negatively affect cognitive function, or aphasia as judged by an inability to communicate by phone. For our analyses, we included all participants with available sleep data at baseline. All participants provided written informed consent.

### Randomization and Blinding

Participants were stratified by stroke status (1 vs ≥2 stroke events) and randomly allocated to: (1) multicomponent EX; (2) ENRICH; or (3) an active control group consisting of stretching and toning exercises (BAT) with an allocation ratio of 2:2:3 (EX:ENRICH:BAT, respectively) using permuted blocks within each stratum. The allocation ratio accounts for the 2 planned contrasts using the Dunnett test (27). Allocation was concealed.

Assessors were blinded to participants’ allocation and participants were asked to refrain from discussing their study involvement or experience during assessments with assessors. Participants and those who delivered the interventions were not blinded.

### Sample Size Calculation

The sample size was powered to evaluate the treatment effect between-groups on the Alzheimer’s Disease Assessment Scale Plus (ADAS-Cog-Plus) at the end of the 6-month intervention (25). We assumed a standardized effect size of 0.6 of EX on cognitive function based on our prior work using the 11-item ADAS-Cog (28). Assuming an alpha of 0.05, 39 participants per group (ie, total sample of 117) would provide a power greater than 0.80. We then assumed a standardized effect size of 0.7 for the ADAS-Cog-Plus, given it has greater sensitivity to changes in cognition compared with either the 11- or 13-item ADAS-Cog (29). After accounting for 15% attrition and 2:2:3 allocation, 34 participants were randomized to EX, 34 to ENRICH, and 52 to BAT, for a total sample of 120.

### Interventions

Each treatment arm included twice-weekly supervised classes of 60 minutes over 6 months and have been described previously (25). All instructors were trained by the research team over a 3-hour session and delivered the interventions based on written protocols.

EX was a multicomponent, individually progressed intervention based on the Fitness and Mobility Exercise program (www.fameexercise.com) (30). It included strength training, aerobic, agility, and balance exercises. Intensity of aerobic training was monitored by the Borg Rating of Perceived Exertion (RPE) and heart rate monitors. Participants were asked initially to complete the exercise sessions at a RPE of 12 (fairly light to somewhat difficult) and were progressed to a maximum target RPE of 16 (difficult to very difficult).

ENRICH included computerized cognitive training (31), other activities that used apps, and others that were based on improvisation and mental activities from the PERK activities program (32). It was designed based on a prior pilot study and current evidence (14,33). Each class began with participants being asked to memorize a 7-item word list. Next, participants completed approximately 15 minutes of the brain-training program Lumosity using an individually issued iPad; these games become progressively more challenging with better performance. The remaining 30 minutes of class time were spent completing a variety of social games and mental activities in pairs or as a class. Some of these activities used apps on the iPad (eg, Heads-Up, Teledoodle), and others were based on improvisation and mental activities from the PERK program (32). Improvisation and mental activities from PERK were group-based and included by design in order to promote interactions and socialization among participants. At the end of the class, participants were asked to recall as many of the words from the word list issued at the beginning of class.

BAT consisted of stretches, deep breathing and relaxation exercises, general posture education, grip strength and dexterity exercises, and light isometric toning exercises (34). Once a month, an education seminar replaced other activities.

### Measures

We report measures acquired at baseline, 6 months (ie, end of intervention), and 12 months (ie, 6-month follow-up). All assessments were conducted by blinded assessors.

The Functional Comorbidity Index measured the number of comorbid conditions (35). Global cognitive function was assessed by MMSE (26) and the Montreal Cognitive Assessment (MoCA) (36). The Center for Epidemiologic Studies-Depression scale was assessed for depressive symptoms (37). Motor function of the upper and lower extremities was assessed by the Fugl-Meyer Assessment Motor scale (38). Participants also self-reported OSA diagnosis at baseline, as well as physical activity using the Community Health Activities Model Program for Seniors (CHAMPS) (39).

#### Cognitive function

Our primary outcome was the ADAS-Cog-Plus (29). The ADAS-Cog-Plus uses a multidimensional item response theory model to generate a global cognitive score from the 13-item ADAS-Cog (ADAS-Cog-13) (40) and additional standard cognitive assessments. We used the ADAS-Cog-13, Trail Making Test Parts A and B, Digit Span Forward and Backward, Animal Fluency, and Vegetable Fluency as the input variables into the scoring algorithm. These tests collectively assess key cognitive domains commonly affected by stroke—memory, attention, and executive functions. Lower ADAS-Cog Plus scores represent better cognitive performance; specifically, scores of approximately −1.0 indicate healthy cognitive functioning, of 0.0 indicate mild cognitive impairment, and of 1.0 indicate dementia (29). We included the ADAS-Cog-13 as a secondary outcome (40), wherein a change of 3.0 points is a minimally important difference (41).

A computerized version of the Stroop task was also included as a secondary outcome (42). The task measures the response inhibition and selective attention components of executive function. Participants completed the task using the program E-prime on a Windows-based computer and Cedrus RB-540 response pad. Color (eg, RED, BLUE) and noncolor (eg, DISK, SCREEN) words appeared individually on the screen with 2 000 ms duration and were printed in 1 of 3 colors (ie, blue, green, or yellow). Participants were instructed to press the response pad button, which was the same color as the font color of the word as quickly and accurately as possible. Following 18 practice trials, the task consisted of 42 neutral trials (eg, the word DISK printed in green font), 42 congruent trials (eg, the word GREEN printed green font), and 42 incongruent trials (eg, word GREEN printed in blue font) presented in random order. We then calculated each participant’s Stroop Interference Ratio as the incongruent median reaction time (in milliseconds) minus the median response time for congruent trials, divided by the congruent median reaction time—using only trials with correct responses (42). Higher scores are indicative of a stronger Stroop effect, and thus, poorer executive function.

#### Device-measured sleep quantity and quality

Device-measured sleep quantity and quality were indexed using the MotionWatch8 (MW8), a uni-axial, wrist-worn accelerometer with evidence of validity and reliability (43,44). We used 60-second epochs, which is consistent with current guidelines for estimating sleep (45).

At baseline, participants were fitted with the MW8 and provided detailed information on its features (ie, the light sensor, event marker button, and status indicator). Participants were instructed to press the event marker button each night when they started trying to sleep and again each morning when they finished trying to sleep. The established protocol for wrist-worn actigraphy suggests participants wear the MW8 on the nondominant wrist for a period of 14 days (45); however, this protocol was modified for older adults with stroke such that if the nondominant side was the stroke-affected side, then we placed the MW8 on the dominant wrist.

Participants were also given the 9-item Consensus Sleep Diary (CSD) and asked to complete it each morning upon waking (46). The responses from the CSD were used to confirm sleep windows identified by participants (44). In cases where the event marker and CSD entry disagreed for the start time of the sleep window, we used activity cessation and light sensor data from the MW8 to determine “lights out.” Similarly, when the event marker and CSD entry disagreed for the end of the sleep window, we used activity onset and “lights on” to determine the end of the sleep window. If responses from the CSD entry disagreed with the event markers entered by participants as the start of the day (ie, finished trying to sleep and awake and out of bed), we used activity onset and light sensor data to determine the start of the day. Similarly, when the event marker and CSD entry disagreed for the end of day (ie, time spent trying to sleep), we used activity cessation and light sensor data to determine the end of the day.

Details of our data reduction procedure have been published (44). Briefly, data were analyzed using MotionWare 1.0.27 (cam*n*tech). Data prior to recorded wake-time on the first full day of recording were manually removed in order to only investigate full 24-hour recordings of activity. Each day of activity consisted of when the participant self-reported being awake and out of bed. Participant self-report was confirmed via event marker time stamps from MW8. The MotionWare software was then used to estimate sleep duration (ie, total time spent sleeping) and sleep efficiency (ie, the proportion of time spent sleeping versus the amount of time spent trying to sleep, expressed as a percentage).

#### Self-reported sleep quality

We measured self-reported sleep quality using the Pittsburgh Sleep Quality Index (PSQI) (47). The questionnaire surveys sleep quality spanning the previous month and has good evidence of validity and reliability (47).

### Statistical Analyses

We performed all statistical analyses in R version 4.1.2 using the *psych, lme4,* and *lsmeans* packages. Our statistical code and output are available on GitHub (https://github.com/ryanfalck/Vitality-Baseline-Sleep-Moderation-Analysis). All models followed the intention-to-treat principle.

We classified participants as having good or poor: (1) device-measured sleep duration; (2) device-measured sleep efficiency; or (3) self-reported sleep quality at baseline. We indexed participants as having good baseline sleep duration as an average sleep duration 420–490 minutes per night at baseline (48); poor baseline sleep duration was categorized as anything outside of this range. Good baseline sleep efficiency was indexed as an average nightly efficiency ≥85% (49), whereas good baseline self-reported sleep quality was categorized as a PSQI score ≤5 (47). Participants who failed to meet either the sleep efficiency or self-reported sleep quality criterion were classified as having poor efficiency or self-reported quality, respectively. Each of these criteria was chosen based on current guidelines for healthy sleep or epidemiological methods for classifying good versus poor sleepers (47–49).

We then examined if treatment effects for our primary outcome, ADAS-Cog-Plus, were moderated by baseline device-measured sleep duration or efficiency or self-reported quality categorization (good vs poor) using linear mixed models with restricted maximum likelihood estimation. The model included random intercepts, and fixed effects of time at baseline, end of the intervention, and 6-month follow-up, group assignment (ie, EX, ENRICH, and BAT), and their interaction. We conducted 3 separate models to address each sleep metric (ie, device-measured sleep duration or sleep efficiency, or self-reported sleep quality); each model included baseline sleep categorization (ie, good vs poor) as a fixed effect, as well as its interaction with time and group assignment (ie, sleep categorization × time × group). Baseline ADAS-Cog-Plus, MMSE score, Fugl-Meyer motor score, and OSA diagnosis at baseline were included as fixed-effect covariates; these covariates were used in the primary outcome paper (4). Unequal variance was allowed across time, group, and sleep categorization. Estimated marginal means were calculated for each treatment group at baseline, end of the intervention, and 6-month follow-up based on sleep categorization.

We then performed planned contrasts using the Dunnett test (27), a multiple comparison procedure, to assess differences in ADAS-Cog-Plus at the end of the intervention and 6-month follow-up between: (1) EX versus BAT and (2) ENRICH versus BAT. The overall alpha was set at 0.05. Each contrast was estimated separately for good and poor baseline sleep categorizations. In the event of significant effects, we conducted post hoc contrasts comparing the estimated between-group differences in ADAS-Cog-Plus for participants categorized as having good versus poor baseline sleep (ie, good sleep – poor sleep). This was done in order to determine if baseline sleep categorization significantly moderated the effect of a given intervention.

Linear mixed models with restricted maximum likelihood estimation were also conducted on our secondary outcomes of ADAS-Cog-13 and Stroop Interference Ratio. Baseline value of outcome, MMSE score, Fugl-Meyer motor score, and OSA diagnosis at baseline were included as fixed-effect covariates. Estimated marginal means were then calculated for each intervention group at baseline, end of the intervention, and 6-month follow-up based on sleep categorization. Planned contrasts using the Dunnett test were then used to assess differences in outcome at the end of the intervention and 6-month follow-up, stratified by baseline sleep categorization. For significant effects, we then conducted post hoc contrasts comparing the estimated between-group differences in ADAS-Cog-13 based on baseline sleep categorization (ie, good sleep – poor sleep). Given the exploratory nature of our analysis, we did not control for multiple comparisons.

We then conducted a separate sensitivity analysis, wherein we examined whether baseline self-reported physical activity level affected our results. For each of our models, we included CHAMPS measured physical activity as an additional covariate.

## Results

Figure 1 illustrates our CONSORT diagram. One hundred and twenty participants were enrolled and randomized from June 6, 2014 to February 26, 2019. The final measurements were made on March 3, 2020, and were not affected by the COVID-19 pandemic. Of the 120 participants randomized to this study, 109 participants (EX = 29; ENRICH = 31; BAT = 49) completed 7 days observation of device-measured sleep at baseline. All 120 participants (EX = 34; ENRICH = 34; BAT = 52) completed the PSQI at baseline. The attrition rate was 14% at the end of the 6-month intervention, and 20% at the end of the 6-month follow-up.

The mean baseline age of participants was 71 years (*SD* = 9) and 62% were male (Table 1). Mean baseline ADAS-Cog-Plus score of 0.22 (*SD* = 0.80), indicating that participants had cognitive impairment (29). The mean baseline Fugl-Meyer Assessment Motor score of 81.21/100 (*SD* = 23.85) indicates moderate to mild motor impairment (50). Seventeen participants (EX = 2; ENRICH = 3; BAT = 12) self-reported OSA diagnosis at baseline.

**Table 1.** Participant Characteristics

	EX (*N* = 34)	ENRICH (*N* = 34)	BAT (*N* = 52)
Age	70.65 (9.14)	71.29 (9.25)	70.44 (7.81)
Males *n*, %	21, 61.8%	23, 67.6%	30, 57.7%
Body mass index (kg/m^2^)	27.37 (3.69)	27.32 (4.45)	27.99 (5.05)
Level of education *n*, %			
High school of less	6, 17.6%	5, 14.7%	13, 26.5%
Trade school or some university	10, 29.4%	8, 23.5%	18, 36.7%
University degree or higher	18, 52.9%	21, 61.8%	21, 40.4%
Number of strokes	1.09 (0.29)	1.15 (0.50)	1.29 (0.72)
Type of stroke			
Hemorrhagic	8, 23.5%	10, 29.4%	15, 28.8%
Ischemic	22, 64.7%	18, 52.9%	33, 63.5%
Other	4, 11.7%	6, 17.6%	4, 7.6%
Hemisphere affected by stroke			
Left	16, 47.1%	14, 41.2%	31, 59.6%
Right	17, 50.0%	18, 52.9%	16, 30.8%
Bilateral	1, 2.9%	0, 0.0%	3, 5.7%
Unknown	0, 0.0%	2, 5.9%	2, 3.8%
Obstructive sleep apnea diagnosis	2, 5.9%	3, 8.8%	12, 23.1%
Functional Comorbidity Index	3.44 (2.05)	2.97 (1.49)	3.67 (1.78)
Instrumental activities of daily living	6.82 (1.83)	7.00 (1.26)	6.77 (1.78)
Mini-Mental State Examination	27.15 (2.56)	27.56 (2.52)	27.15 (2.40)
Montreal Cognitive Assessment	21.26 (3.70)	22.82 (3.99)	21.69 (4.49)
Center for Epidemiological Studies-Depression scale	9.59 (6.42)	8.35 (10.30)	9.92 (7.79)
Fugl-Meyer Motor score	73.25 (26.48)	80.09 (24.57)	87.38 (19.91)
Alzheimer’s Disease Assessment Scale-Cognitive Plus	0.39 (0.77)	0.12 (0.71)	0.17 (0.88)
13-Item Alzheimer’s Disease Assessment scale	18.16 (7.47)	16.42 (6.29)	17.19 (8.00)
Stroop Interference Ratio^*^	0.15 (0.10)	0.15 (0.11)	0.16 (0.14)
Motionwatch sleep duration (min/night)^†^	413.46 (60.86)	434.11 (77.58)	433.70 (69.68)
Motionwatch sleep efficiency (%)^*^	83.29 (8.11)	84.94 (7.86)	85.79 (6.69)
Pittsburgh Sleep Quality Index	6.00 (3.09)	6.38 (3.30)	5.88 (2.83)

*Notes*:

^*^Stroop Interference Ratio is calculated as: incongruent median reaction time (in milliseconds) − congruent median reaction time (in milliseconds) / congruent median reaction time (in milliseconds); lower scores indicate less interference, or better performance.

^†^Means and *SD*s based on subset of 109 participants (EX = 29; ENRICH = 31; BAT = 49) with Motionwatch measured sleep at baseline.

Participant baseline characteristics stratified by device-measured sleep duration and sleep efficiency, and self-reported sleep categorizations are described in Supplementary Materials S1A–C, respectively. Of the 109 participants with device-measured sleep at baseline, we classified 13/29 EX, 17/31 ENRICH, and 28/49 BAT participants as having good device-measured sleep duration at baseline; 15/29 EX, 17/31 ENRICH, and 32/49 BAT participants were classified as having good device-measured efficiency. For self-reported sleep quality, 16/34 EX, 16/34 ENRICH, and 28/52 BAT participants were classified as having good self-reported sleep quality.

### Treatment Moderating Effects of Device-Measured Baseline Sleep Duration

We describe estimated marginal means for each group based on baseline sleep categorization in Table 2. Between-group differences (ie, EX vs BAT and ENRICH vs BAT) are described in Table 3. For participants in either EX or ENRICH with good baseline sleep duration, there were no significant differences in cognitive performance from BAT participants with good baseline sleep duration at either the end of the intervention or at 6-month follow-up. There were no effects of EX or ENRICH on ADAS-Cog-Plus, ADAS-Cog-13, or Stroop Interference Ratio compared with BAT at either the end of the intervention or at 6-month follow-up for participants with poor baseline sleep duration.

**Table 2.** Estimated Marginal Means ± Standard Errors for Changes in Cognitive Function and Physical Function at Baseline, 6 Months (End of Intervention) and 12 Months (ie, 6-Month Follow-Up) by Treatment Group and Sleep Quality

		Baseline^*^			6 Months			12 Months		
	Outcome	EX	ENRICH	BAT	EX	ENRICH	BAT	EX	ENRICH	BAT
Good Sleep Duration (420–490 min/night)	ADAS-Cog-Plus	0.76 (0.96)	0.03 (0.54)	0.27 (1.06)	−0.20 ± 0.16	−0.26 ± 0.13	0.05 ± 0.11	−0.27 ± 0.17	−0.37 ± 0.13	−0.11 ± 0.11
	ADAS-Cog 13	21.56 (9.92)	15.25 (4.99)	18.57 (8.64)	13.80 ± 1.52	13.67 ± 1.23	15.91 ± 1.08	12.39 ± 1.70	12.11 ± 1.23	12.87 ± 1.08
	Stroop Interference Ratio	0.16 (0.14)	0.20 (0.11)	0.15 (0.18)	0.16 ± 0.03	0.14 ± 0.03	0.16 ± 0.02	0.16 ± 0.04	0.17 ± 0.03	0.15 ± 0.02
Poor Sleep Duration (<420 or >490 min/night)	ADAS-Cog-Plus	0.25 (0.72)	0.05 (0.74)	0.11 (0.76)	−0.31 ± 0.11	−0.04 ± 0.12	−0.15 ± 0.09	−0.20 ± 0.11	−0.02 ± 0.12	−0.16 ± 0.09
	ADAS-Cog 13	16.86 (6.51)	16.57 (7.23)	16.36 (7.62)	12.21 ± 1.07	14.56 ± 1.16	14.38 ± 0.85	12.91 ± 1.09	14.91 ± 1.19	13.36 ± 0.89
	Stroop Interference Ratio	0.15 (0.09)	0.11 (0.09)	0.16 (0.12)	0.14 ± 0.02	0.14 ± 0.03	0.15 ± 0.02	0.13 ± 0.02	0.13 ± 0.03	0.16 ± 0.02
Good Sleep Efficiency (≥85%)	ADAS-Cog-Plus	0.22 (0.80)	−0.05 (0.43)	0.09 (0.87)	−0.15 ± 0.13	−0.26 ± 0.12	−0.13 ± 0.08	−0.08 ± 0.13	−0.34 ± 0.11	−0.19 ± 0.08
	ADAS-Cog 13	16.35 (7.29)	14.94 (5.48)	16.36 (6.97)	13.89 ± 1.22	13.73 ± 1.15	14.69 ± 0.79	14.09 ± 1.29	12.23 ± 1.15	12.65 ± 0.79
	Stroop Interference Ratio	0.15 (0.11)	0.16 (0.12)	0.16 (0.17)	0.18 ± 0.03	0.14 ± 0.02	0.17 ± 0.02	0.18 ± 0.03	0.16 ± 0.02	0.17 ± 0.02
Poor Sleep Efficiency (<85%)	ADAS-Cog-Plus	0.61 (0.82)	0.15 (0.85)	0.32 (0.90)	−0.38 ± 0.12	0.01 ± 0.12	0.08 ± 0.12	−0.34 ± 0.13	−0.01 ± 0.13	0.02 ± 0.13
	ADAS-Cog 13	20.43 (8.16)	17.34 (7.21)	18.83 (9.72)	11.73 ± 1.19	14.83 ± 1.21	15.96 ± 1.20	11.81 ± 1.23	15.26 ± 1.24	14.72 ± 1.29
	Stroop Interference Ratio	0.17 (0.09)	0.14 (0.10)	0.15 (0.09)	0.12 ± 0.03	0.13 ± 0.03	0.13 ± 0.03	0.11 ± 0.03	0.14 ± 0.03	0.13 ± 0.03
Good Subjective Sleep Quality (PSQI ≤5)	ADAS-Cog-Plus	0.57 (0.90)	0.25 (0.72)	0.18 (0.95)	−0.25 ± 0.13	−0.14 ± 0.12	−0.18 ± 0.10	−0.07 ± 0.13	−0.11 ± 0.13	−0.16 ± 0.10
	ADAS-Cog 13	20.10 (7.59)	18.08 (6.55)	17.56 (8.26)	13.72 ± 1.21	13.78 ± 1.18	14.04 ± 0.93	14.24 ± 1.24	13.03 ± 1.21	13.11 ± 0.93
	Stroop Interference Ratio	0.13 (0.10)	0.13 (0.12)	0.14 (0.17)	0.15 ± 0.03	0.16 ± 0.03	0.15 ± 0.02	0.14 ± 0.03	0.13 ± 0.03	0.16 ± 0.02
Poor Subjective Sleep Quality (PSQI >6)	ADAS-Cog-Plus	0.24 (0.61)	0.00 (0.71)	0.16 (0.81)	−0.33 ± 0.12	−0.20 ± 0.11	0.04 ± 0.09	−0.29 ± 0.12	−0.27 ± 0.11	−0.13 ± 0.10
	ADAS-Cog 13	16.44 (7.14)	14.94 (5.85)	16.75 (7.83)	11.68 ± 1.12	14.17 ± 1.10	15.81 ± 0.91	12.03 ± 1.17	13.74 ± 1.10	13.10 ± 0.95
	Stroop Interference Ratio	0.17 (0.10)	0.17 (0.10)	0.18 (0.11)	0.15 ± 0.03	0.11 ± 0.02	0.16 ± 0.02	0.14 ± 0.03	0.17 ± 0.02	0.15 ± 0.02

*Notes*: ADAS-Cog-Plus = Alzheimer’s Disease Assessment Scale Plus; ADAS-Cog 13 = Alzheimer’s Assessment Scale Cognitive 13-item.

All models controlled for baseline Mini-Mental State Exam score, Fugl*-*Meyer motor score, obstructive sleep apnea diagnosis, and baseline outcome.

Stroop Interference Ratio is calculated as: incongruent median reaction time (in ms) − congruent median reaction time (in ms) / congruent median reaction time (in milliseconds); lower scores indicate less interference, or better performance.

^*^Mean (*SD*).

**Table 3.** Estimated Mean Differences and 95% Confidence Intervals for Between-Group Differences at 6 Months (ie, End of Intervention) and 12 Months (ie, 6-Month Follow-Up)

		Adjusted Between-Group Differences at 6 Months (95% CI)				Adjusted Between-Group Differences at 12 Months (95% CI)			
	Outcome	EX vs BAT	*p* Value	ENRICH vs BAT	*p* Value	EX vs BAT	*p* Value	ENRICH vs BAT	*p* Value
Good Sleep Duration (420–490 min/night)	ADAS-Cog-Plus	−0.25 (−0.67, 0.17)	.307	−0.31 (−0.67, 0.04)	.095	−0.16 (−0.61, 0.28)	.623	−0.26 (−0.62, 0.09)	.178
	ADAS-Cog 13	−2.12 (−6.15, 1.92)	.399	−2.24 (−5.72, 1.24)	.262	−0.48 (−4.85, 3.89)	.944	−0.77 (−4.25, 2.71)	.825
	Stroop Interference Ratio	−0.01 (−0.09, 0.08)	.983	−0.02 (−0.10, 0.05)	.690	0.01 (−0.09, 0.11)	.961	0.02 (−0.06, 0.10)	.822
Poor Sleep Duration (<420 or >490 min/night)	ADAS-Cog-Plus	−0.15 (−0.46, 0.15)	.430	0.12 (−0.21, 0.44)	.628	−0.05 (−0.36, 0.27)	.907	0.13 (−0.20, 0.47)	.559
	ADAS-Cog 13	−2.17 (−5.14, 0.81)	.187	0.18 (−2.97, 3.32)	.982	−0.45 (−3.49, 2.60)	.910	1.55 (−1.69, 4.79)	.459
	Stroop Interference Ratio	−0.01 (−0.08, 0.06)	.922	−0.02 (−0.09, 0.05)	.779	−0.03 (−0.10, 0.03)	.424	−0.04 (−0.11, 0.03)	.406
Good Sleep Efficiency (≥85%)	ADAS-Cog-Plus	−0.02 (−0.34, 0.30)	.972	−0.14 (−0.44, 0.17)	.517	0.11 (−0.22, 0.45)	.658	−0.15 (−0.45, 0.16)	.470
	ADAS-Cog 13	−0.81 (−3.92, 2.31)	.775	−0.96 (−3.99, 2.06)	.689	1.45 (−1.81, 4.70)	.507	−0.41 (−3.43, 2.61)	.921
	Stroop Interference Ratio	0.01 (−0.06, 0.08)	.932	−0.03 (−0.09, 0.04)	.590	0.01 (−0.07, 0.08)	.968	−0.01 (−0.08, 0.05)	.856
Poor Sleep Efficiency (<85%)	ADAS-Cog-Plus	−0.47 (−0.84, −0.09)	.012	−0.08 (−0.45, 0.29)	.836	−0.36 (−0.75, 0.03)	.080	−0.03 (−0.42, 0.36)	.968
	ADAS-Cog 13	−4.23 (−7.91, −0.55)	.021	−1.13 (−4.78, 2.53)	.705	−2.91 (−6.80, 0.97)	.171	0.54 (−3.33, 4.40)	.918
	Stroop Interference Ratio	−0.01 (−0.09, 0.07)	.959	0.01 (−0.07, 0.08)	.984	0.01 (−0.08, 0.09)	.717	−0.03 (−0.11, 0.06)	.965
Good Subjective Sleep Quality (PSQI ≤5)	ADAS-Cog-Plus	−0.06 (−0.41, 0.28)	.874	0.04 (−0.30, 0.38)	.929	0.10 (−0.25, 0.44)	.751	0.06 (−0.29, 0.40)	.886
	ADAS-Cog 13	−0.32 (−3.61, 2.97)	.955	−0.27 (−3.54, 3.00)	.966	1.14 (−2.21, 4.48)	.659	−0.07 (−3.40, 3.25)	.996
	Stroop Interference Ratio	0.00 (−0.07, 0.07)	.999	0.01 (−0.06, 0.08)	.934	−0.02 (−0.09, 0.05)	.768	−0.03 (−0.11, 0.04)	.452
Poor Subjective Sleep Quality (PSQI >6)	ADAS-Cog-Plus	−0.37 (−0.69, −0.05)	.021	−0.24 (−0.56, 0.08)	.161	−0.16 (−0.50, 0.18)	.456	−0.14 (−0.46, 0.18)	.515
	ADAS-Cog 13	−4.13 (−7.22, −1.04)	.006	−1.64 (−4.69, 1.41)	.382	−1.06 (−4.30, 2.18)	.677	0.65 (−2.45, 3.76)	.837
	Stroop Interference Ratio	−0.02 (−0.09, 0.05)	.820	−0.05 (−0.12, 0.02)	.159	−0.01 (−0.08, 0.06)	.906	0.02 (−0.05, 0.08)	.799

*Notes*: ADAS-Cog-Plus = Alzheimer’s Disease Assessment Scale Plus; ADAS-Cog 13 = Alzheimer’s Assessment Scale Cognitive 13-item.

All models controlled for baseline Mini-Mental State Exam score, Fugl−Meyer motor score, obstructive sleep apnea diagnosis, and baseline outcome.

Stroop Interference Ratio is calculated as: incongruent median reaction time (in ms) − congruent median reaction time (in ms) / congruent median reaction time (in ms); lower scores indicate less interference, or better performance.

### Treatment Moderating Effects of Device-Measured Baseline Sleep Efficiency

Among participants classified with good baseline sleep efficiency, there were no effects of EX or ENRICH on cognitive performance at either the end of the intervention or at 6-month follow-up. Following the end of the intervention, participants in EX classified with poor baseline sleep efficiency had significantly better ADAS-Cog-Plus (estimated mean difference: −0.47; 95% CI [−0.84, −0.09]; *p* = .012) than BAT participants with poor baseline sleep efficiency. There were no between-group differences at 6-month follow-up. We also determined that EX participants with poor baseline sleep efficiency had significantly better ADAS-Cog-13 performance (estimated mean difference: −4.23; 95% CI [−7.91, −0.55]; *p* = .021) than BAT participants with poor baseline sleep efficiency following the end of the intervention; there were no between-group differences at 6-month follow-up. There was no effect of EX on Stroop Interference Ratio, irrespective of sleep efficiency at baseline. There were no effects of ENRICH at either the end of the intervention or 6-month follow-up compared with BAT for participants with poor baseline sleep efficiency.

Post hoc, we determined that there was a significant difference in the effects of EX versus BAT on ADAS-Cog-Plus between baseline sleep efficiency categories following the end of the intervention (estimated mean difference: 0.44; 95% CI [0.02, 0.86]; *p* = .041), whereby EX participants categorized with poor baseline sleep efficiency had significantly greater improvement in ADAS-Cog-Plus performance than EX with good baseline sleep efficiency. The effects of EX versus BAT on the ADAS-Cog-13 were not significantly different between baseline sleep efficiency categories at the end of the intervention (estimated mean difference: 3.42; 95% CI [−0.73, 7.58]; *p* = .105).

### Treatment Moderating Effects of Baseline Self-Reported Sleep Quality

There were no effects of EX or ENRICH on ADAS-Cog-Plus or ADAS-Cog-13 at either the end of the intervention or at 6-month follow-up for participants with good baseline self-reported sleep quality. Among participants with poor baseline sleep quality, EX had significantly better ADAS-Cog-Plus (estimated mean difference: −0.37; 95% CI [−0.69, −0.05]; *p = *.021) and ADAS-Cog-13 performance (estimated mean difference: −4.13; 95% CI [−7.22, −1.04]; *p* = .006) than BAT at the end of the intervention. There were no effects of EX on Stroop Interference Ratio, regardless of baseline sleep efficiency categorization. Among participants with poor baseline self-reported sleep quality, there were no effects of ENRICH on cognitive performance compared with BAT at either the end of the intervention or 6-month follow-up. Post hoc, there were no significant differences between baseline self-reported sleep quality categories in the effects of EX versus BAT on either ADAS-Cog-Plus (estimated mean difference: 0.31; 95% CI [−0.10, 0.71]; *p* = .133) or ADAS-Cog-13 (estimated mean difference: 3.81; 95% CI [−0.08, 7.70]; *p = *.055) at the end of the intervention.

### Sensitivity Analyses

The results of our sensitivity analyses are described in Supplementary Material S2. Including baseline physical activity as a covariate did not affect the strength or direction of our results.

## Discussion

We show that adults with chronic stroke and poor sleep may experience greater improvements in cognitive function from EX than their peers with good quality sleep. Sleep does not appear to moderate the effects of ENRICH on cognitive function in adults with chronic stroke.

Among participants classified as having either poor device-measured sleep efficiency (ie, <85% efficiency) or poor self-reported sleep quality (PSQI >6) at baseline, EX had significantly better performance on both the ADAS-Cog-Plus and the ADAS-Cog-13 at trial completion compared with those in BAT. Importantly, EX with poor baseline sleep efficiency improved on the ADAS-Cog-13 by 4.23 points compared with BAT with poor baseline sleep efficiency; EX with poor baseline self-reported sleep quality improved by 4.13 points compared with BAT with poor baseline self-reported sleep quality. Each of these improvements exceeds the established minimally clinically important difference (ie, ≥3.0 points) (41). Post hoc comparisons indicated EX categorized with poor baseline sleep efficiency had a significantly greater improvement in ADAS-Cog-Plus performance than EX with good baseline sleep efficiency. Neither good (ie, 420–490 minutes per night) nor poor baseline sleep duration (<420 minutes per night or >490 minutes per night) moderated the effects of EX on cognitive function.

We did not find that any measure of baseline sleep moderated the effects of EX on cognitive performance at 6-month follow-up. As noted in our protocol (25) and our primary outcome paper (4), the primary endpoint for this trial was 6 months (ie, the end of the intervention), with participants followed for an additional 6 months (ie, 6-month follow-up). Our results indicate that baseline sleep quality moderated the effects of EX such that there was an effect at the end of the 6-month intervention for individuals with poor sleep quality, but not those with good sleep quality. However, once the intervention concluded, there was no moderating effect of baseline sleep on the effects of EX on cognitive function at 6-month follow-up.

For ENRICH, baseline sleep did not moderate the effects of the intervention on cognitive function. In the primary analysis of this study, the effects of ENRICH on cognitive function were null (4), In spite of this null effect, it is still unclear why sleep would not moderate the effects of ENRICH. Sleep is critical to performance in multiple domains of cognitive function (51), including learning and memory (52). We thus suggest more work is needed to clarify whether or not baseline sleep moderates the effects of ENRICH on cognitive function.

Interestingly, our results align with those of a secondary analysis of a RCT by Sewell and colleagues (24). The authors examined whether an EX intervention improved sleep among cognitively healthy older adults. Although the intervention did not improve sleep, the authors did find that participants with poor self-reported sleep quality at baseline had significantly greater improvements in cognitive function following 6 months of twice-weekly aerobic EX compared with their peers with good self-reported sleep quality. Sewell and colleagues (24) did not find that self-reported sleep duration moderated the effects of EX on cognition. Our results found that both baseline self-reported sleep quality and device-measured sleep efficiency moderate the effects of EX on cognitive function in older adults with chronic stroke, whereas sleep duration did not moderate the effects of EX on cognition—suggesting a similar phenomenon to that first indicated by Sewell and colleagues (24).

The implications of sleep quantity and quality on cognitive health are still under investigation. Sleep duration represents the total quantity of sleep incurred over the course of the night, whereas sleep efficiency is a measure of sleep quality (53); the PSQI is a self-reported measure of sleep quality (47). A meta-analysis by Xu and colleagues (54) determined that both long and short sleep durations were associated with an increased risk of cognitive impairment. The authors also noted that poor sleep efficiency was associated with a 15% increased risk of cognitive impairment, although poor subjective sleep quality was not associated with risk of cognitive impairment. Indeed, the current available evidence examining the impact of self-reported sleep quality on cognitive function in older adults has produced mixed results such that the field is moving toward device-based measured estimates of sleep quality (eg, actigraphy-measured sleep efficiency) (55). Our results highlight that sleep quality as indexed by actigraphy-measured sleep efficiency, and confirmed by self-reported sleep quality, moderates the effects of EX on cognitive function. Neither device-measured nor self-reported sleep duration was a moderator. This suggests that sleep quality, but not sleep quantity, might be a key moderator of the effects of EX on cognitive function. Although our findings should be treated with caution, given that this is a secondary analysis of a 6-month RCT, the results we found seem to further suggest that individuals with poor sleep quality may be a key target population for using EX as a therapy to promote cognitive function.

Current hypotheses suggest EX and sleep may affect cognitive function through both shared and divergent pathways (23). Several cross-sectional studies have determined a moderating relationship of sleep on the association of physical activity and multiple domains of cognition in healthy older adults (56–58). A cross-sectional study of healthy older adults by Wilckens and colleagues (59) suggested a mediating relationship of sleep on the association between physical activity and cognition—specifically in the domains of executive function, verbal fluency, memory, and processing speed. Our own research group cross-sectionally determined that there were independent relationships of physical activity and sleep with healthy older adult cognition as measured by the ADAS-Cog Plus (60). However, these previous works should be treated cautiously within the context of our own findings. Our study is a secondary analysis of a RCT and thus provides causal evidence that sleep moderates the effects of exercise on cognition in older adults with chronic stroke, unlike these cross-sectional studies, which can only suggest associations. Additionally, each of these cross-sectional studies was conducted in healthy older adults without stroke. Older adults with chronic stroke have significantly worse cognitive performance and sleep, and are significantly less active than their nonstroke peers (22,61). Having a stroke also causes fundamental changes in brain structure and function, which may elicit substantial changes in sleep (62); although poor sleep in stroke survivors is likely due to multiple issues, including: (1) enhanced vulnerability for OSA and sleep-wake cycle disorders (22); (2) changes in oscillations of cortical networks that can affect sleep (62); (3) physical consequences of stroke such as chronic pain, restricted mobility, and reduced physical activity (63,64); and (4) psychological changes such as depression and fatigue (65).

Experimental data examining the mechanisms by which EX and sleep interact on cognition are scarce. In a mouse model by Zielinski and colleagues (66), the authors determined that there were significant benefits of EX on spatial memory under normal sleep conditions, but not under chronic moderate sleep restriction; a similar pattern was found for hippocampal cell activation and brain-derived neurotrophic factor concentration in the hippocampus. Unfortunately, we are unaware of any other evidence examining how sleep moderates the effects of EX on cognitive function, and thus highlight that there is a substantial need to untangle how EX and sleep interact with cognitive health.

### Clinical Implications

Our findings highlight the clinical importance of baseline sleep as a moderator of the effects of EX on cognition. Notably, older adult stroke survivors in the EX intervention with poor actigraphy-measured sleep efficiency (ie, <85% efficiency) or poor self-reported sleep quality (PSQI >6) at baseline improved on the ADAS-Cog-13 by amounts that exceeded the established minimal clinically important difference (ie, ≥3.0 points) (41). Clinicians who aim to improve the cognitive health of older adult stroke survivors using EX should thus also monitor the sleep of their patients, as it may have important implications on the effects of EX. Monitoring sleep using commercial devices such as a fitness tracker like FitBit or Apple Watch might provide valuable information about the sleep quality of their patients; however, even simple questionnaires, like the PSQI, might provide a useful index of sleep quality. At this time, it is difficult to speculate as to whether or not EX promotes cognitive function in older adult stroke survivors with good sleep quality. Nonetheless, we highlight that there are substantial benefits of EX for older adults with chronic stroke irrespective of sleep quality, including physical function, cardiovascular health, and health-related quality of life (67–69). We thus suggest at this time that EX should be considered a frontline strategy to promote cognitive health for all older adults with chronic stroke, and EX may be especially beneficial for individuals with poor sleep quality.

### Limitations

This was a secondary analysis wherein we stratified our results based on sleep categorization, and thus treatment groups are unbalanced at baseline. Although we used the Dunnett test to adjust for comparisons of 2 treatment groups to the control (ie, EX vs BAT and ENRICH vs BAT), we did not further adjust for multiple comparisons for each sleep categorization due to the exploratory nature of this study and because our stratified treatment groups were not fully powered. Our sample had heterogeneous stroke types and locations, which may be linked to different sleep disturbances and cognitive issues.

There is not yet criterion evidence of validity for the MW8 among adults with chronic stroke; in a previous investigation (22), we determined that MW8 provides reliable estimates of sleep duration and efficiency among older adults with and without cognitive impairment. We queried whether participants were diagnosed with OSA, but it is still plausible that some participants had undiagnosed OSA. We did not query about continuous positive air pressure device use. Other sleep disorders, which we did not query about (eg, restless leg syndrome) could have also confounded our results. The study sample only included chronic stroke survivors with mild-to-moderate motor impairments.

Due to the diverse content of the ENRICH intervention, there may have been insufficient dose and specificity of training to elicit an effect. In addition, although EX was an individually progressed exercise program, not all components of ENRICH became progressively more challenging.

## Conclusions

The findings from this secondary analysis of a RCT suggest that baseline sleep quality may moderate the effects of EX on cognitive function in people with chronic stroke, and can induce clinically important improvements in cognitive performance in this population at risk for dementia. Adults with chronic stroke and poor sleep may potentially be a target population for promoting cognitive health through EX.

## Supplementary Material

glae264_suppl_Supplementary_Materials

## Data Availability

De-identified participant data will be made available by the corresponding author to others who propose a reasonable scientific request and obtain appropriate ethics.
