# Supplementary material for: Does Sleep Moderate the Effects of Exercise Training or Complex Mental and Social Activities on Cognitive Function in Adults With Chronic Stroke? Secondary Analysis of a Randomized Trial
Source: J Gerontol A Biol Sci Med Sci. 2024 Nov 8;80(1):glae264. doi: 10.1093/gerona/glae264 (PMC11632229; doi:10.1093/gerona/glae264)
Supplement: glae264_suppl_Supplementary_Materials [file glae264_suppl_supplementary_materials.pdf]

Supplementary Material S1. Participant baseline characteristics stratified by MotionWatch8-measured A) sleep duration and B) efficiency, and C) self-reported sleep quality categorizations

Supplementary Material S1A. Participant characteristics stratified by device-measured sleep duration categorizations

	Good Sleep Duration			Bad Sleep Duration		
	EX (N=9)	ENRICH (N=13)	BAT (N=19)	EX (N=20)	ENRICH (N=18)	BAT (N=30)
Age	72.22 (13.26)	73.00 (9.26)	73.37 (7.18)	70.60 (8.11)	70.17 (9.67)	68.27 (8.00)
Males n, %	5, 55.6%	7, 53.8%	9, 47.4%	12, 60.0%	14, 77.8%	20, 66.7%
Body Mass Index (kg/m2)	26.93 (2.11)	26.61 (4.52)	28.34 (3.25)	27.52 (3.79)	28.38 (4.00)	27.87 (6.13)
University Degree or Higher n, %	7, 77.8%	10, 76.9%	6, 31.6%	9, 45.0%	10, 55.6%	12, 40.0%
Number of Strokes	1.22 (0.44)	1.15 (0.55)	1.26 (0.56)	1.00 (0.00)	1.11 (0.47)	1.27 (0.78)
Type of Stroke						
Hemorrhagic	2, 22.2%	5, 38.5%	6, 31.6%	5, 25.0%	5, 27.8%	9, 30.0%
Ischemic	6, 66.7%	8, 61.5%	11, 57.9%	13, 65.0%	7, 38.9%	19, 63.3%
Other	1, 11.1%	0, 0.0%	2, 10.5%	2, 10.0%	6, 33.3%	2, 6.6%
Hemisphere Affected by Stroke						
Left	3, 33.3%	5, 38.5%	10, 52.6%	9, 45.0%	7, 38.9%	19, 63.3%
Right	6, 66.7%	8, 61.5%	7, 36.8%	10, 50.0%	10, 55.6%	9, 30.0%
Bilateral	0, 0.0%	0, 0.0%	1, 5.3%	1, 5.0%	0, 0.0%	1, 3.3%
Unknown	0, 0.0%	0, 0.0%	1, 5.3%	0, 0.0%	1, 5.6%	1, 3.3%
Mini Mental State Exam	26.89 (3.02)	28.54 (1.45)	27.05 (2.50)	27.25 (2.47)	26.94 (2.98)	27.20 (2.43)
Fugl-Meyer Motor Score	79.11 (21.35)	81.62 (26.38)	90.24 (20.04)	71.74 (26.29)	80.76 (21.99)	84.26 (20.55)
ADAS-Cog Plus	0.76 (0.96)	0.03 (0.54)	0.27 (1.06)	0.25 (0.72)	0.05 (0.74)	0.11 (0.76)
ADAS-Cog 13	21.56 (9.92)	15.25 (4.99)	18.57 (8.64)	16.86 (6.51)	16.57 (7.23)	16.36 (7.62)
MW8 Duration (min/night)	455.41 (19.20)	458.18 (22.79)	445.67 (17.78)	394.59 (63.99)	416.73 (97.42)	426.12 (87.68)
MW8 Efficiency (%)	86.14 (4.97)	90.16 (5.63)	85.74 (6.45)	82.01 (9.00)	81.17 (7.13)	85.83 (6.95)
PSQI	5.89 (3.66)	7.31 (2.66)	5.89 (2.38)	6.50 (3.03)	6.00 (3.73)	5.87 (2.97)

[1]Participants categorized based on median sleep duration (Good Sleep Duration ≥ 420 min/night; Bad Sleep Duration < 420 min/night)

ADAS-Cog Plus: Alzheimer's Disease Assessment Scale Cognitive Plus; ADAS-Cog 13: 13-item Alzheimer's Disease Assessment Scale; MW8: Motionwatch8 Actigraphy; PSQI: Pittsburgh Sleep Quality Index

Supplementary Material S1B. Participant characteristics stratified by device-measured sleep efficiency categorizations

	Good Sleep Efficiency[1]			Bad Sleep Efficiency		
	EX (N=15)	ENRICH (N=17)	BAT (N=32)	EX (N=14)	ENRICH (N=14)	BAT (N=17)
Age	71.40 (11.50)	72.35 (10.36)	70.41 (7.11)	70.79 (8.47)	70.14 (8.44)	69.94 (9.74)
Males n, %	10, 66.7%	10, 58.8%	17, 53.1%	7, 50.0%	11, 78.6%	12, 70.6%
Body Mass Index (kg/m2)	26.90 (2.64)	27.48 (4.99)	27.73 (4.33)	27.80 (4.00)	27.82 (3.30)	28.64 (6.57)
University Degree or Higher n, %	10, 66.7%	12, 70.6%	13, 40.6%	6, 42.9%	8, 57.1%	5, 29.4%
Number of Strokes	1.13 (0.35)	1.24 (0.66)	1.25 (0.62)	1.00 (0.00)	1.00 (0.00)	1.29 (0.85)
Type of Stroke						
Hemorrhagic	5, 33.3%	5, 29.4%	9, 28.1%	2, 14.3%	5, 35.7%	6, 35.3%
Ischemic	8, 53.3%	10, 58.8%	20, 62.5%	11, 78.6%	5, 35.7%	10, 58.8%
Other	2, 13.4%	2, 11.8%	3, 9.3%	1, 7.1%	4, 28.6%	1, 5.9%
Hemisphere Affected by Stroke						
Left	6, 40.0%	6, 35.3%	18, 56.2%	6, 42.9%	6, 42.9%	11, 64.7%
Right	9, 60.0%	10, 58.8%	12, 37.5%	7, 50.0%	8, 57.1%	4, 23.5%
Bilateral	0, 0.0%	0, 0.0%	1, 3.1%	1, 7.1%	0, 0.0%	1, 5.9%
Unknown	0, 0.0%	1, 5.9%	1, 3.1%	0, 0.0%	0, 0.0%	0, 0.0%
Mini Mental State Exam	28.00 (2.48)	28.41 (1.50)	27.47 (2.20)	26.21 (2.49)	26.64 (3.23)	26.53 (2.79)
Fugl-Meyer Motor Score	72.29 (21.49)	75.62 (25.07)	86.13 (19.25)	75.93 (28.18)	87.43 (20.79)	87.50 (23.23)
ADAS-Cog Plus	0.22 (0.80)	-0.05 (0.43)	0.09 (0.87)	0.61 (0.82)	0.15 (0.85)	0.32 (0.90)
ADAS-Cog 13	16.35 (7.29)	14.94 (5.48)	16.36 (6.97)	20.43 (8.16)	17.34 (7.21)	18.83 (9.72)
MW8 Duration (min/night)	427.29 (60.07)	461.85 (65.59)	448.97 (73.67)	398.65 (60.29)	400.43 (79.79)	404.97 (52.05)
MW8 Efficiency (%)	89.48 (2.98)	90.58 (3.84)	89.88 (3.24)	76.66 (6.37)	78.09 (5.69)	78.10 (4.20)
PSQI	7.40 (3.58)	6.00 (2.87)	5.50 (2.24)	5.14 (2.28)	7.21 (3.83)	6.59 (3.43)

[1]Participants categorized based MW8 Sleep Efficiency (Good Sleep Efficiency ≥ 85% MW8 Sleep Efficiency; Bad Sleep Efficiency < 85% MW8 Sleep Efficiency)

ADAS-Cog Plus: Alzheimer's Disease Assessment Scale Cognitive Plus; ADAS-Cog 13: 13-item Alzheimer's Disease Assessment Scale; MW8: Motionwatch8 Actigraphy; PSQI: Pittsburgh Sleep Quality Index

Supplementary Material S1C. Participant characteristics stratified by self-reported sleep quality categorizations

	Good Sleep Quality[1]			Bad Sleep Quality		
	EX (N=16)	ENRICH (N=16)	BAT (N=28)	EX (N=18)	ENRICH (N=18)	BAT (N=24)
Age	72.50 (9.08)	70.88 (9.27)	69.04 (6.16)	69.00 (9.12)	71.67 (9.49)	72.04 (9.27)
Males n, %	11, 68.8%	12, 75.0%	21, 75.0%	10, 55.6%	11, 61.1%	9, 37.5%
Body Mass Index (kg/m2)	27.74 (3.48)	27.17 (4.61)	28.16 (4.21)	27.05 (3.93)	27.46 (4.42)	27.79 (5.98)
University Degree or Higher n, %	6, 37.5%	10, 62.5%	12, 42.9%	12, 66.7%	11, 61.1%	9, 37.5%
Number of Strokes	1.12 (0.34)	1.31 (0.70)	1.36 (0.87)	1.06 (0.24)	1.00 (0.00)	1.21 (0.51)
Type of Stroke						
Hemorrhagic	2, 12.5%	5, 31.2%	11, 39.3%	6, 33.3%	5, 27.8%	4, 16.7%
Ischemic	12, 75.0%	7, 43.8%	17, 60.7%	10, 55.6%	11, 61.1%	16, 66.7%
Other	2, 12.5%	4, 25.0%	0, 0.0%	2, 11.2%	2, 11.2%	4, 16.7%
Hemisphere Affected by Stroke						
Left	10, 62.5%	7, 43.8%	16, 57.1%	6, 33.3%	7, 38.9%	15, 62.5%
Right	5, 31.2%	8, 50.0%	10, 35.7%	12, 66.7%	10, 55.6%	6, 25.0%
Bilateral	1, 6.2%	0, 0.0%	2, 7.1%	0, 0.0%	0, 0.0%	1, 4.2%
Unknown	0, 0.0%	1, 6.2%	0, 0.0%	0, 0.0%	1, 5.6%	2, 8.3%
Mini Mental State Exam	26.25 (2.77)	27.06 (2.41)	26.96 (2.70)	27.94 (2.13)	28.00 (2.61)	27.38 (2.04)
Fugl-Meyer Motor Score	74.69 (30.68)	72.36 (24.73)	91.24 (9.86)	71.81 (22.44)	86.11 (23.37)	83.00 (26.82)
ADAS-Cog Plus	0.57 (0.90)	0.25 (0.72)	0.18 (0.95)	0.24 (0.61)	0.00 (0.71)	0.16 (0.81)
ADAS-Cog 13	20.10 (7.59)	18.08 (6.55)	17.56 (8.26)	16.44 (7.14)	14.94 (5.85)	16.75 (7.83)
MW8 Duration (min/night)	417.83 (59.12)	417.27 (77.81)	446.31 (79.30)	409.92 (63.94)	447.99 (76.90)	418.23 (53.46)
MW8 Efficiency (%)	81.83 (6.26)	84.34 (9.62)	86.10 (6.73)	84.48 (9.39)	85.44 (6.34)	85.42 (6.78)
PSQI	3.38 (1.26)	3.50 (1.03)	3.82 (1.22)	8.33 (2.22)	8.94 (2.34)	8.29 (2.18)

[1]Participants categorized based PSQI Score (Good Sleep Quality ≤ 5 PSQI score; Bad Sleep Quality < 6 PSQI Score)

ADAS-Cog Plus: Alzheimer's Disease Assessment Scale Cognitive Plus; ADAS-Cog 13: 13-item Alzheimer's Disease Assessment Scale; MW8: Motionwatch8 Actigraphy; PSQI: Pittsburgh Sleep Quality Index

Supplementary Material S2. Sensitivity analyses for models including physical activity level as a covariate

Estimated mean differences and 95% confidence intervals for between-group differences at 6 (i.e., end of the intervention) and 12 months (i.e., 6-month follow-up) for all outcomes when including Community Health Activity Monitoring Program for Seniors (CHAMPS) physical activity questionnaire as a covariate

		**Adjusted Between-Group Differences at 6 months (95% CI)**				**Adjusted Between-Group Differences at 12 months (95% CI)**			
	Outcome	EX vs. BAT	p	ENRICH vs. BAT	p	EX vs. BAT	p	ENRICH vs. BAT	p
Good Sleep Duration (420-490 min/night)	ADAS-Cog-Plus	-0.27 (-0.68, 0.15)	0.271	-0.33 (-0.69, 0.03)	0.076	-0.18 (-0.63, 0.26)	0.558	-0.28 (-0.64, 0.08)	0.147
	ADAS-Cog 13	-2.07 (-6.12, 1.98)	0.417	-2.17 (-5.68, 1.34)	0.289	-0.40 (-4.80, 3.99)	0.960	-0.69 (-4.20, 2.82)	0.854
	Stroop Interference Score	0.00 (-0.09, 0.09)	0.998	-0.02 (-0.10, 0.06)	0.774	0.01 (-0.09, 0.11)	0.917	0.02 (-0.06, 0.10)	0.772
Poor Sleep Duration (<420 or >490 min/night)	ADAS-Cog-Plus	-0.15 (-0.45, 0.16)	0.453	0.12 (-0.20, 0.44)	0.619	-0.04 (-0.36, 0.27)	0.923	0.14 (-0.19, 0.47)	0.550
	ADAS-Cog 13	-2.21 (-5.20, 0.78)	0.179	0.17 (-2.99, 3.33)	0.983	-0.49 (-3.55, 2.57)	0.896	1.54 (-1.71, 4.79)	0.466
	Stroop Interference Score	-0.01 (-0.08, 0.06)	0.911	-0.02 (-0.09, 0.05)	0.777	-0.03 (-0.10, 0.03)	0.404	-0.04 (-0.11, 0.03)	0.399
Good Sleep Efficiency (≥85%)	ADAS-Cog-Plus	-0.03 (-0.35, 0.29)	0.962	-0.14 (-0.45, 0.17)	0.510	0.11 (-0.23, 0.45)	0.690	-0.15 (-0.46, 0.16)	0.464
	ADAS-Cog 13	-0.74 (-3.86, 2.39)	0.805	-0.93 (-3.95, 2.10)	0.708	1.55 (-1.72, 4.82)	0.466	-0.37 (-3.40, 2.65)	0.933
	Stroop Interference Score	0.01 (-0.06, 0.08)	0.870	-0.02 (-0.09, 0.04)	0.620	0.01 (-0.06, 0.09)	0.901	-0.01 (-0.08, 0.05)	0.880
Poor Sleep Efficiency (<85%)	ADAS-Cog-Plus	-0.46 (-0.84, -0.08)	0.014	-0.08 (-0.46, 0.29)	0.828	-0.35 (-0.75, 0.04)	0.086	-0.03 (-0.42, 0.36)	0.964
	ADAS-Cog 13	-4.34 (-8.03, -0.65)	0.018	-1.07 (-4.73, 2.59)	0.727	-3.04 (-6.94, 0.86)	0.150	0.57 (-3.30, 4.44)	0.910
	Stroop Interference Score	-0.01 (-0.09, 0.07)	0.931	0.01 (-0.07, 0.08)	0.967	-0.03 (-0.11, 0.06)	0.648	0.01 (-0.08, 0.09)	0.970
Good Subjective Sleep Quality (PSQI≤5)	ADAS-Cog-Plus	-0.04 (-0.39, 0.30)	0.934	0.05 (-0.29, 0.39)	0.912	0.11 (-0.23, 0.46)	0.677	0.06 (-0.28, 0.41)	0.872
	ADAS-Cog 13	-0.34 (-3.67, 2.99)	0.951	-0.27 (-3.56, 3.01)	0.965	1.12 (-2.26, 4.50)	0.671	-0.08 (-3.42, 3.27)	0.996
	Stroop Interference Score	-0.01 (-0.08, 0.07)	0.983	0.01 (-0.06, 0.08)	0.945	-0.02 (-0.10, 0.05)	0.704	-0.04 (-0.11, 0.04)	0.431
Poor Subjective Sleep Quality (PSQI>6)	ADAS-Cog-Plus	-0.39 (-0.71, -0.07)	0.015	-0.26 (-0.58, 0.06)	0.127	-0.18 (-0.52, 0.16)	0.377	-0.16 (-0.48, 0.16)	0.446
	ADAS-Cog 13	-4.12 (-7.23, -1.01)	0.007	-1.63 (-4.70, 1.44)	0.392	-1.05 (-4.31, 2.21)	0.686	0.66 (-2.46, 3.79)	0.835
	Stroop Interference Score	-0.01 (-0.08, 0.06)	0.885	-0.05 (-0.11, 0.02)	0.188	-0.01 (-0.08, 0.06)	0.956	-0.02 (-0.05, 0.09)	0.769

All models controlled for baseline Mini Mental State Exam score, Fugl-Meyer motor score, obstructive sleep apnea diagnosis, baseline CHAMPS physical activity energy expenditure (kcal/week), and baseline outcome

Stroop Interference Ratio is calculated as: incongruent median reaction time (in milliseconds) − congruent median reaction time (in milliseconds) / congruent median reaction time (in milliseconds); lower scores indicate less interference, or better performance.

ADAS-Cog-Plus: Alzheimer's Disease Assessment Scale Plus; ADAS-Cog 13: Alzheimer's Assessment Scale Cognitive 13-item
